# Poly(octamethylene citrate) Modified with Glutathione as a Promising Material for Vascular Tissue Engineering

**DOI:** 10.3390/polym15051322

**Published:** 2023-03-06

**Authors:** Agata Flis, Martina Trávníčková, Filip Koper, Karolina Knap, Wiktor Kasprzyk, Lucie Bačáková, Elżbieta Pamuła

**Affiliations:** 1 Department of Biomaterials and Composites, Faculty of Materials Science and Ceramics, AGH University of Science and Technology, 30 Mickiewicza Ave., 30-059 Kraków, Poland; 2 Laboratory of Biomaterials and Tissue Engineering, Institute of Physiology of the Czech Academy of Sciences, Vídeňská 1083, 142 20 Prague, Czech Republic; 3 Department of Biotechnology and Physical Chemistry, Faculty of Chemical Engineering and Technology, Cracow University of Technology, 24 Warszawska St., 31-155 Kraków, Poland

**Keywords:** poly(alkylene citrates), poly(1,8-octametylene citrate), citric acid, vascular tissue engineering, glutathione (GSH), cytocompatibility, vascular smooth-muscle cells (VSMCs), adipose tissue-derived stem cells (ASCs)

## Abstract

One of the major goals of vascular tissue engineering is to develop much-needed materials that are suitable for use in small-diameter vascular grafts. Poly(1,8-octamethylene citrate) can be considered for manufacturing small blood vessel substitutes, as recent studies have demonstrated that this material is cytocompatible with adipose tissue-derived stem cells (ASCs) and favors their adhesion and viability. The work presented here is focused on modifying this polymer with glutathione (GSH) in order to provide it with antioxidant properties, which are believed to reduce oxidative stress in blood vessels. Cross-linked poly(1,8-octamethylene citrate) (cPOC) was therefore prepared by polycondensation of citric acid and 1,8-octanediol at a 2:3 molar ratio of the reagents, followed by in-bulk modification with 0.4, 0.8, 4 or 8 wt.% of GSH and curing at 80 °C for 10 days. The chemical structure of the obtained samples was examined by FTIR-ATR spectroscopy, which confirmed the presence of GSH in the modified cPOC. The addition of GSH increased the water drop contact angle of the material surface and lowered the surface free energy values. The cytocompatibility of the modified cPOC was evaluated in direct contact with vascular smooth-muscle cells (VSMCs) and ASCs. The cell number, the cell spreading area and the cell aspect ratio were measured. The antioxidant potential of GSH-modified cPOC was measured by a free radical scavenging assay. The results of our investigation indicate the potential of cPOC modified with 0.4 and 0.8 wt.% of GSH to produce small-diameter blood vessels, as the material was found to: (i) have antioxidant properties, (ii) support VSMC and ASC viability and growth and (iii) provide an environment suitable for the initiation of cell differentiation.

## 1. Introduction

Vascular tissue engineering continues to be one of the most rapidly advancing fields of biomedicine [1]. Numerous techniques have been developed to replace irreversibly damaged blood vessels, such as the use of autologous patient blood vessels, allografts from other human donors, xenografts from animal donors, synthetic polymeric vascular prostheses, and grafts created by methods of tissue engineering, e.g., on the basis of synthetic or biological scaffolds and cells, preferably autologous patient cells [1]. In some cases, the polymeric scaffolds can be chemically modified to provide them with specific properties, e.g., a supportive effect on the cell adhesion that facilitates the subsequent growth of cells and their differentiation towards the desired cell phenotypes present in vascular tissue [1]. Currently, damaged blood vessels can be replaced with the use of various polymer materials, depending on the diameter of the vessels [2]. Prostheses made of poly(ethylene terephthalate) (PET), polyurethane and expanded polytetrafluoroethylene (ePTFE) are mainly used for large- and medium-diameter blood vessels (>6 mm) [2,3,4,5]. The development of small-diameter (<6 mm) prosthetic vascular grafts continues to be a goal of cardiovascular research. One of the main challenges of artificial vascular grafts is the risk of thrombosis due to platelet adhesion and activation, caused by poor patency and by the formation of blood clots [6]. Additionally, the slower blood flow through a small-diameter vessel and the collapse of the vessel walls lead to occlusion of the vessel [4,7]. Both PET and ePTFE have mechanical properties differing from those of autologous blood vessels, which may also contribute to intimal hypertrophy, i.e., thickening of the *tunica intima* of a blood vessel, as a complication of vascular remodeling [8,9]. It is suggested that the properties of an implanted blood vessel should be similar to those of the native blood vessel, as poorly matched mechanical parameters may hamper the correct adaptation of the implant [1,10,11]. Long-term patency of artificial small-diameter blood vessels (<6 mm) has not yet been achieved [12,13]. A desirable biomaterial feature for blood vessel prostheses is susceptibility to surface modification with various types of biological molecules. These molecules should be able to counteract non-specific protein adsorption processes that may induce an undesirable cellular response, and to promote cell adhesion and growth in a controllable manner [14]. In addition, it is very important to be able to store the biomaterial under normal conditions for long periods of time [15]. The preparation and sterilization of the prosthesis should also be as easy and as quick as possible [15].

Recently, biomaterials based on citric acid, i.e., poly(alkylene citrates) (PACs), have gained attention in many fields of biomedicine, mainly in tissue engineering, but also in drug delivery systems, bioimaging, biosensing and cancer therapy [16,17,18]. Their mechanical properties and their degradability can be controlled by adjusting the molar ratio of citric acid to diol, by changing the type of diol, as well as by changing the temperature and atmosphere of cross-linking [14,16,19]. Their optical and catalytic properties can also be controlled [20]. Increasing the temperature and/or the duration of the cross-linking process increases the bond density and improves the mechanical strength of the material, while at the same time it can reduce its flexibility and slow down its degradation rate [21]. These materials are also susceptible to many surface modification techniques to improve their biological properties, including cell adhesion [22]. Cross-linking of PAC results in the formation of a biodegradable material with a certain amount of unreacted functional groups (free carboxyl and hydroxyl groups) [14,21]. This is a key property in designing materials with the desired properties (e.g., antioxidant, adhesive, antibacterial and fluorescent) [21]. 

Incorporating ascorbic acid into the PAC structure improves its ability to get rid of free radicals and increases its hydrophilicity (due to the additional hydroxyl group from the attached compound), while it does not affect the mechanical and degradation properties. Moreover, the antioxidative properties are present during the entire polymer degradation process [23]. The participation of azide and alkyne functional groups in diols during cross-linking of the polymer adds an additional mechanism of thermal reaction, which increases the tensile strength of the material [24]. Other cross-linking mechanisms based on UV irradiation or on redox mechanisms can be added by incorporating monomers containing unsaturated chemical groups (e.g., vinyl). Thanks to this strategy, the preserved carboxylic and hydroxyl groups can be used to connect other, e.g., bioactive, molecules [23]. Introducing nanoparticles based on compounds such as polylactide, poly(lactide-*co-*glycolide), hydroxyapatite, multi-walled carbon nanotubes or modified silica increases the tensile strength and the Young’s modulus of poly(octamethylene citrate) (POC) biomaterials [21,25,26]. Through the reaction of α-amino acid with citric acid and aliphatic diols, PACs with fluorescent properties can be obtained [18,27]. This material shows a high quantum efficiency of 62.3%, and the choice of appropriate amino acids allows the fluorescence signal that is obtained to be adjusted to the expected wavelength (corresponding to the colors from blue to red) [28,29]. This approach enables these materials to be used in the fields of bioimaging and drug delivery [21].

Moreover, the surface of PACs has been demonstrated to be compatible with various types of cells, e.g., human aortic smooth-muscle cells and human aortic endothelial cells, which is essential for the purposes of vascular tissue engineering [17,20]. An advantage of PACs is their easy functionalization. Most of the currently used biomaterials require additional processing for further modification [14]. Due to their flexibility, strength, hemocompatibility and ability to release nitric oxide (i.e., an important gaseous signaling molecule promoting vasodilatation and endothelial cell growth, and preventing graft thrombosis and restenosis), citric acid-based biomaterials have been tested for a variety of vascular applications, including surface modification of commercially available vascular grafts [21]. 

In this paper, we focus on cross-linked poly(1,8-octamethylene citrate) (cPOC), obtained as a result of the polycondensation reaction of citric acid and 1,8-octanediol [16,17,19,20]. Citric acid is a non-toxic metabolic product (an important component of the Krebs cycle) and can be used as a cheap and easily available substrate [14,17]. It is also a reactive monomer that can form hydrogen bonds in the polyester network [14,17]. In addition, 1,8-octanediol enables the formation of ester bonds with citric acid. It is also the aliphatic diol with the highest molecular weight and is soluble in water and non-toxic at the same time [17]. Our recent biological studies performed on cPOC extracts showed that changing the molar ratio of reagents during synthesis (from the 1:1 ratio commonly described in the literature to a ratio of 2:3) had a positive effect on the viability and proliferation of adipose tissue-derived stem cells (ASCs), while maintaining the required physicochemical properties of the biomaterial [30]. 

The aim of the work presented here was to modify cPOC with glutathione (GSH), i.e., a low molecular weight thiol synthesized by most organisms to protect them against oxidative stress [31,32]. Incorporating GSH into cPOC, which, to the best of our knowledge, has not been carried out before, would endow it with antioxidant properties. Oxidative stress reduction is especially important for protecting blood vessels from inflammation and from the formation of cholesterol plaques. GSH also has many other functions in the human body, including those related to metabolism, transport, catalysis, formation of deoxyribonucleic acids, stabilization of protein thiol groups, maintenance of the reduced form of other molecules (e.g., cysteine, coenzyme A, ascorbic acid), as well as the prevention of non-enzymatic glycations of proteins, e.g., during diabetes or ageing [32]. It has been demonstrated that citric acid can react with GSH to produce a highly fluorescent compound, while biological tests have proved that this compound is non-cytotoxic and can be further explored in terms of possible biomedical applications [33]. 

The results presented in this paper have allowed us to select the optimal amount of GSH, which would have a beneficial effect on the adhesion and proliferation of cells cultured on the modified cPOC material. The advantage of an in-bulk modification technique of this kind is its low price, simplicity, controllability of the amount of added reagent and the possibility of carrying out the reaction under standard laboratory conditions. The choice of the cells used in the work presented in this paper was justified by the structure of the blood vessel, where vascular smooth-muscle cells (VSMCs) are the main component of its medium part, i.e., *tunica media*. Another cell type chosen for this study was ASCs, which show the ability to differentiate towards VSMCs when cultured in a medium with a specific composition [34].

## 2. Materials and Methods

### 2.1. Chemicals

Anhydrous citric acid (Alpha Aesar, Haverhill, MA, USA), 1,8-octanediol (Angene, Hyderabad, India), l-glutathione (GSH) (Fluorochem, Glossop, UK), 96% ethanol (POCH, Gliwice, Poland), 2,2-diphenyl-1-picrylhydrazyl (DPPH; Sigma-Aldrich, Merck, Darmstadt, Germany), methanol (POCH, Gliwice, Poland), the In vitro Toxicology Assay Kit (Resazurin based) (Sigma-Aldrich, Merck, Darmstadt, Germany), calcein-AM (Sigma-Aldrich, Merck, Darmstadt, Germany), propidium iodide (Sigma-Aldrich, Merck, Darmstadt, Germany), Dulbecco’s Modified Eagle Medium (DMEM; Gibco, Thermo Fisher Scientific, Waltham, MA, USA), fetal bovine serum (FBS; Gibco, Thermo Fisher Scientific, Waltham, MA, USA), gentamicin (Lek Pharmaceuticals, Ljubljana, Slovenia), phosphate-buffered saline (PBS; Sigma-Aldrich, Merck, Darmstadt, Germany), trypsin-EDTA (Sigma-Aldrich, Merck, Darmstadt, Germany), fibroblast growth factor 2 (FGF2; GenScript Biotech, Piscataway, NJ, USA), bone morphogenetic protein 4 (BMP4; Sigma-Aldrich, Merck, Darmstadt, Germany), transforming growth factor beta 1 (TGFß1; Abcam, Cambridge, UK) and diiodomethane (Sigma-Aldrich, Merck, Darmstadt, Germany) were used. All chemicals and solvents were of analytical grade and were used without additional purification.

### 2.2. Cell Types Used in Experiments

Cells used for biological characterization were: adipose tissue-derived stem cells (ASCs, isolated from lipoaspirate under ethical approval issued by the Ethics Committee at Na Bulovce Hospital in Prague, and under informed consent obtained from the patient [34]) and vascular smooth-muscle cells (VSMCs, isolated from porcine aorta by the explantation method [34]). For the experiments, both cell types were used in passage 3.

### 2.3. Synthesis and Modification of Poly(octamethylene citrate)

#### 2.3.1. Synthesis of Prepolymers

Poly(octamethylene citrate) (POC) prepolymers were prepared, as described elsewhere [30]. In brief, anhydrous citric acid and 1,8-octanediol were mixed at a molar ratio of 2:3 in glass vials (9.34 g of citric acid and 10.66 g of diol). The reactants were then heated at 140 °C for 40 min on a magnetic stirrer (IKA, CMAG HS7/10, Warsaw, Poland; 100 rpm). The resulting reaction mixtures were dissolved in 40 mL of 96% ethanol, were precipitated in 150 mL of deionized water, centrifuged (MPW-350, Warsaw, Poland; 5000 rpm, 10 min) and freeze-dried (CHRIST Alpha 2–4 LDplus, Osterode am Harz, Germany; 0.35 mbar, −85 °C). After freeze-drying, the prepolymers were dissolved in 96% ethanol to obtain 30% *w/v* solutions.

#### 2.3.2. Modification and Cross-Linking of Prepolymers

The aqueous GSH solutions of 50 mg/mL and 100 mg/mL concentrations were prepared by adding the GSH powder to deionized water and thoroughly mixing. The prepolymer modification process was carried out by adding an appropriate amount of GSH aqueous solution (50 mg/mL or 100 mg/mL) to 100 mL of the 30% *w/v* prepolymer solutions (specified in Table 1) and casting the obtained mixtures on polypropylene or polystyrene oval forms, or directly into 96-well plates (for biological assessment). The last step involved cross-linking the samples in forms, performed by curing them at 80 °C for 10 days, the first day under atmospheric pressure; nd the remaining days under reduced pressure (Memmert, UF 55, 200 mbar). The final percentages of GSH in the cross-linked materials were 0.4, 0.8, 4 or 8 wt.%. Unmodified polymer, i.e., cPOC, served as a control (Table 1).

### 2.4. FTIR-ATR Spectroscopy

The chemical structure of the samples was examined by attenuated total reflectance Fourier-transform infrared spectroscopy (ATR-FTIR) using a Bruker Tensor 27 spectrometer with a diamond crystal (Bruker, San Jose, CA, USA). The measurements were carried out in the mid-infrared range (4000–600 cm^−1^) at 64 scans per sample with a resolution of 4 cm^−1^. The obtained spectra were analyzed using OPUS 8.7 SP2 software.

### 2.5. Water Contact Angle and Surface Free Energy

The surface wettability and the surface free energy were studied by measuring the contact angle of deionized water and diiodomethane with a sessile drop method using a DSA 25 Drop Shape Analysis automatic system (Krüss). For each sample, 10 individual measurements were performed, 5 s after the droplet was deposited on the surface. The average droplet size was 0.50 µL. The surface free energy values (ƴ_p_—dispersive part, ƴ_p_—polar part and ƴ_s_—total surface free energy) were determined using the Owens-Wendt method in ADVANCE software.

### 2.6. Antioxidant Properties

The antioxidant activity was determined using a 2,2-diphenyl-1-picrylhydrazyl (DPPH•) free radical scavenging assay. A solution of DPPH• was prepared by adding 15.8 mg of powdered DPPH• to 100 mL of methanol. Then, 120 mg (±1 mg) of each cPOC, cPOC_GSH_0.4 and cPOC_GSH_0.8 sample was weighed and placed into a 24-well plate, and 500 µL of DPPH solution and 100 µL of methanol were added to each well and were allowed to react in a dark place, at room temperature. An empty well with DPPH solution and methanol served as the control. After 2 h, 100 µL of each aliquot was transferred to a 96-well plate. The absorbance values were measured at 517 nm in a spectrophotometer (FLUOstar Omega, BMG LABTECH, Mornington, VIC, Australia). The experiments were performed in triplicate.

### 2.7. Biological Characterization

In vitro cytocompatibility of the cPOCs was performed in direct contact using two different cell types: ASCs and VSMCs. VSMCs were cultured in DMEM, supplemented with 10% FBS and 40 µg/mL of gentamicin. ASCs were cultured in DMEM, supplemented with 10% of FBS, 40 µg/mL of gentamicin and 10 ng/mL of FGF2. After 3 days of ASC culture, 100 µL of DMEM was aspired from each well and was replaced by DMEM with the addition of TGFβ1 (2.5 ng/mL) and BMP4 (2.5 ng/mL), which served as factors differentiating the ASCs towards VSMCs. All cells were cultured in a humidified 5% CO_2_ atmosphere at 37 °C.

Prior to the experiment, cPOC samples unmodified and modified with 0.4, 0.8, 4 and 8 wt.% of GSH were cross-linked for 10 days in 96-well plates, as described in Section 2.2. The materials were sterilized in 70% EtOH (30 min) followed by UV light exposition (30 min) and were rinsed in a cell culture medium for 48 h to remove unreacted monomers which could cause acidification of the cell culture environment. After that, the cell medium was removed, and the samples were allowed to dry. Then, ASCs and VSMCs were seeded and cultured in separate wells at a density of 5 × 10^3^ cells per well in 200 µL of the dedicated cell culture medium at 37 °C in a 5% CO_2_ atmosphere. After 1, 3 and 7 days, the cells were examined under an optical microscope, and pictures were taken to analyze the morphology of cells growing on the material surface. After 3 days, 100 µL of the medium was aspired from each well with ASCs and was replaced with a differentiation medium containing TGFβ1 and BMP4. After 7 days, all cells were fixed with 2% paraformaldehyde and were stained with hematoxylin and eosin.

### 2.8. Cell Morphology and Number Analysis

The pictures of the cells cultured on the polymers were taken with a digital camera coupled with an optical microscope (IX71 Olympus microscope, DP71 digital camera), followed by an analysis of the morphology of the ASCs by means of ImageJ 153e software (https://imagej.net/Fiji, accessed on 27 January 2023). The number of cells was counted, and a total of 100 measurements of both cell area and cell aspect ratio (ratio of the cellular major and minor axes, i.e., the ratio of length to width) were collected using ImageJ software. These measurements were performed only for ASCs (after 1 and 3 days), because the high cell proliferation rate of the VSMCs resulted in a high cell population density, close contact and even multilayered cell growth (i.e., a “hills and valleys” pattern), which made further measurements impossible.

### 2.9. Statistical Analysis

A statistical analysis of the data was performed using a one-way analysis of variance (one-way ANOVA) followed by Tukey’s post hoc test. Hypotheses of normal distribution and equal variance were verified using the Shapiro–Wilk and Levene median tests, respectively (*p* < 0.05). The analyses were performed using OriginPro2022 software. The results were presented as the mean ± standard deviation (SD) for the physicochemical characterization results, or as the standard error of the mean (SE) for the biological characterization results.

## 3. Results

### 3.1. Synthesis, Cross-Linking and Modification of POC

**Figure 1:**
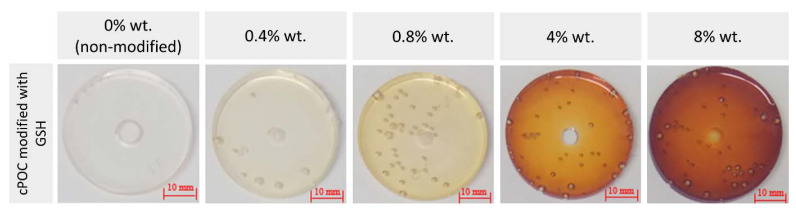
cPOC control sample and cPOC modified with 0.4, 0.8, 4 and 8 wt.% of GSH.

POC was obtained in a polycondensation reaction of citric acid and 1,8-octanediol in a 2:3 molar ratio of the reactants. Before the cross-linking process, the samples were modified with GSH in bulk, which resulted in materials with GSH addition of 0.4 (cPOC_GSH_0.4), 0.8 (cPOC_GSH_0.8), 4 (cPOC_GSH_4) and 8 wt.% (cPOC_GSH_8). The addition of GSH contributed to the yellow–brown color of the samples, and the color intensified with an increase in the amount of GSH modifier (Figure 1). However, the mere change in the color of the modified sample is not sufficient to confirm the evidence of successful incorporation of GSH into the polymer. Preliminary studies of the citric acid reaction with GSH revealed that a part of this tripeptide was hydrolyzed to glutamic acid and dipeptide consisting of glycine and cysteine. Cysteine is capable of reacting with citric acid to form a fluorescent derivative of ring-fused 2-pyridone [35]. It is therefore suspected that a similar reaction might occur during the in-bulk modification of the studied POC materials, and that most of the GSH attached to it in an unchanged form, providing the resulting material with antioxidant properties.

### 3.2. FTIR-ATR Spectroscopy

**Figure 2:**
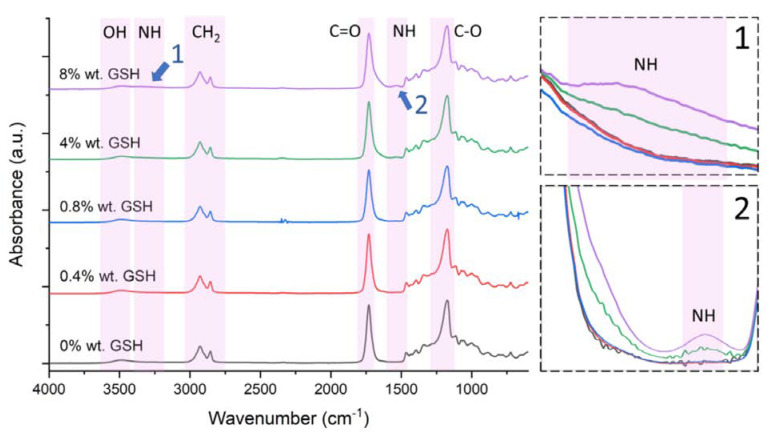
FTIR spectra of cPOC non-modified (0 wt.% of GSH) and modified with 0.4, 0.8, 4 and 8 wt.% of GSH.

The FTIR-ATR spectra (Figure 2) of the GSH-modified cPOC materials confirm the effectiveness of the modification process.

The presence of GSH in the cPOC matrix was confirmed by the appearance of characteristic GSH-derived bands in the infrared spectra. The broad signals in the range of 1650–1500 cm^−1^ were therefore ascribed to the overlapping N–H bending, along with the C–N stretching vibrations of the secondary amide groups of GSH and GSH covalently bound to the polymer backbone or side chains. The broad bands around 3400–3300 cm^−1^ most probably appear as a consequence of the overlapped stretching vibrations of the N–H and O–H vibrational modes [36]. Other bands indicating the presence of GSH in the sample were also detected, but they are less informative because they overlap with the cPOC signals, which dominate the spectra. It is worth pointing out that the intensity of these bands increases with increasing GSH addition. Interestingly, no bands assigned to S–H stretching vibrations were observed around 2550–2600 cm^−1^. This might be associated with the possibility of partial transformation of GSH to disulphide compounds or to derivatives of ring-fused 2-pyridones during cross-linking of the materials. Although signals originating from the thiol groups were not found in the spectra, it is highly probable that some part of the GSH was preserved in a reduced state in the materials, since the samples show substantial antioxidant properties. The absence of specific bands in the spectra might be related to the extremely low concentrations of modifiers used for the synthesis. Nevertheless, the use of FTIR-ATR spectroscopy to study the surface of cPOC materials confirmed the effectiveness of the applied method of synthesizing them and modifying them by adding appropriate volumes of GSH aqueous solution before cross-linking the prepolymer.

### 3.3. Water Contact Angle and Surface Free Energy

**Figure 3:**
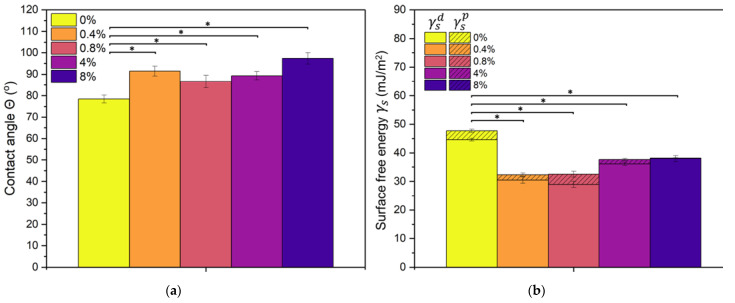
Water contact angle (**a**) and surface free energy values (ƴ_s_^d^—dispersive part, ƴ_s_^p^—polar part) (**b**) of cPOC non-modified (0 wt.%) and modified with 0.4, 0.8, 4 and 8 wt.% of GSH. Mean ± SD, statistically significant differences at * *p* < 0.001.

The increasing amount of GSH in the volume of the cPOC material, in general, resulted in an increased water contact angle (Figure 3a), changing its surface character from slightly hydrophilic to hydrophobic, as evidenced by an increase in the water contact angle from about 80° to almost 100° in the presence of 8 wt.% of GSH. Figure 3b shows the surface free energy values of the cPOC modified with various amounts of GSH. All materials have a significantly higher dispersive part than the polar part of the surface free energy. GSH was found to reduce the surface free energy from 45–50 mJ/m^2^ to 30–40 mJ/m^2^.

### 3.4. Antioxidant Properties

**Figure 4:**
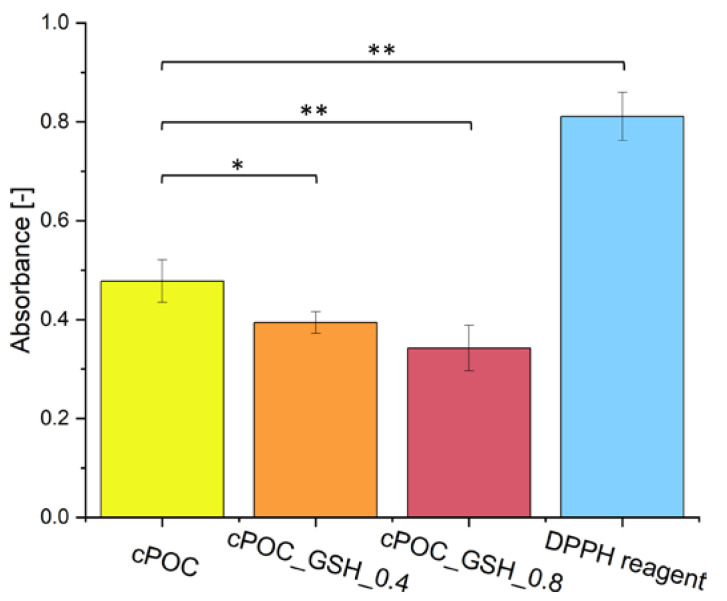
DPPH• free radical scavenging assay results for cPOC, cPOC_GSH_0.4 and cPOC_GSH_0.8. The absorbance value of the DPPH reagent served as a control sample. Mean ± SD, statistically significant differences at * *p* < 0.01 and ** *p* < 0.001.

A DPPH• free radical scavenging assay confirmed the antioxidant properties of the tested samples, as demonstrated by the difference between the absorbance values of the cPOC materials and of the pure DPPH reagent (Figure 4). The lower the absorbance, the stronger the antioxidant effect of the material. Values obtained for cPOC_GSH_0.4 (0.39 ± 0.02) and for cPOC_GSH_0.8 (0.34 ± 0.05) indicated that a greater addition of GSH provides cPOC with better antioxidant properties. An interesting fact is that the absorbance of unmodified cPOC was almost two times lower (0.48 ± 0.04) than the absorbance of the pure DPPH reagent (0.81 ± 0.05). A possible reason for this may be that cPOC itself exhibits antioxidant properties, as already proposed by van Lith et al. [23]. Another potential explanation could be that cPOC materials tend to absorb the DPPH dye from the solution, resulting in a decrease in its absorbance and therefore the appearance of a false positive DPPH test result.

### 3.5. Biological Properties

Our previously published studies [30] demonstrated good cytocompatibility of extracts from cPOC in contact with ASCs. The next step was therefore to check the behavior of cells in direct contact with these materials. The presence of GSH in cPOC materials was expected to provide beneficial antioxidant properties that would support good healing of replacements for damaged blood vessels after they had been implanted into the body. In order to select the optimal amount of GSH in cPOC, ASCs and VSMCs were tested in contact with unmodified and modified materials with 0.4, 0.8, 4 and 8 wt.% of GSH. ASCs and VSMCs were chosen for this study because of their important role in vascular tissue engineering of the main component of the vessel medium part, i.e., the *tunica media*.

**Figure 5:**
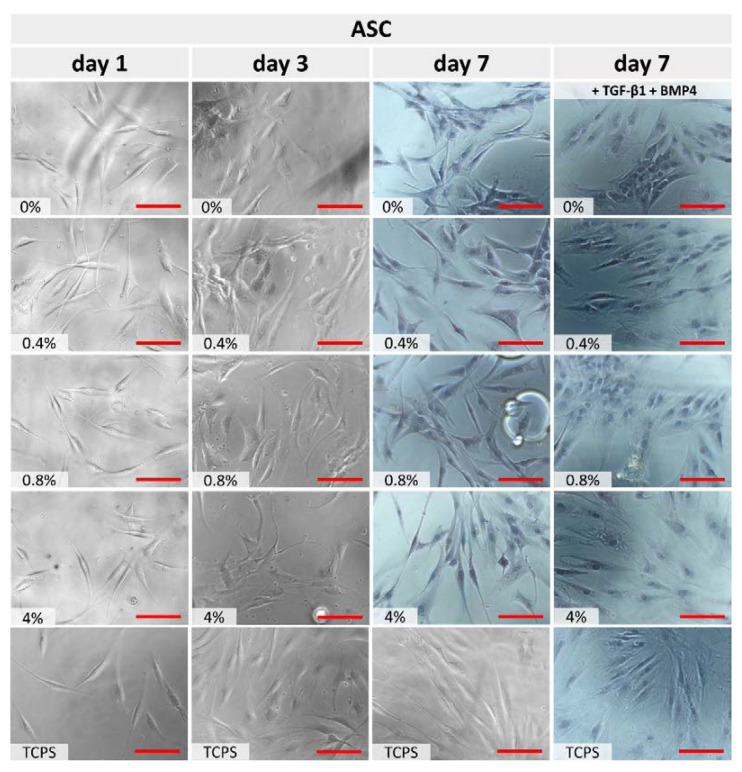
Optical microscopy of ASCs after 1, 3 and 7 days of culture on an unmodified cPOC surface (0 wt.% of GSH) and on cPOC modified with different concentrations of GSH (0.4, 0.8 and 4 wt.%). Cells cultured on TCPS served as the control. After day 7, except TCPS, the cells were stained with hematoxylin and eosin. Scale = 200 μm (red line).

Figure 5 demonstrates ASCs’ grown on the surface of unmodified and modified cPOC materials after 1, 3 and 7 days of culture. Cells cultured on tissue culture polystyrene (TCPS) were used as a control. As expected, the selected materials did not show any cytotoxic effect on this cell type. ASCs, regardless of the concentration of the modifier, grew well on the surface of the materials, and maintained a similar morphology as on the control TCPS. The addition of differentiation factors (TGFβ1 and BMP4) into the medium made the ASC morphology different from that observed in the standard growth medium after 7 days. The cells in the differentiation medium were generally better spread and in closer cell-to-cell contact, starting to form separate islands. Unfortunately, the time range of 7 days (including 4 days after the application of the differentiation medium) was too short to conclude that the cells had undergone differentiation. Moreover, due to the strong autofluorescence of the polymer, it was impossible to perform selective immunofluorescence staining of the cells (e.g., α-actin or calponin staining, confirming the cell differentiation towards VSMCs). However, on the basis of the results, which demonstrated changes in cell shape, it can be concluded that at least some part of the differentiation process may have taken place, and the cPOC materials did not interfere with it.

**Figure 6:**
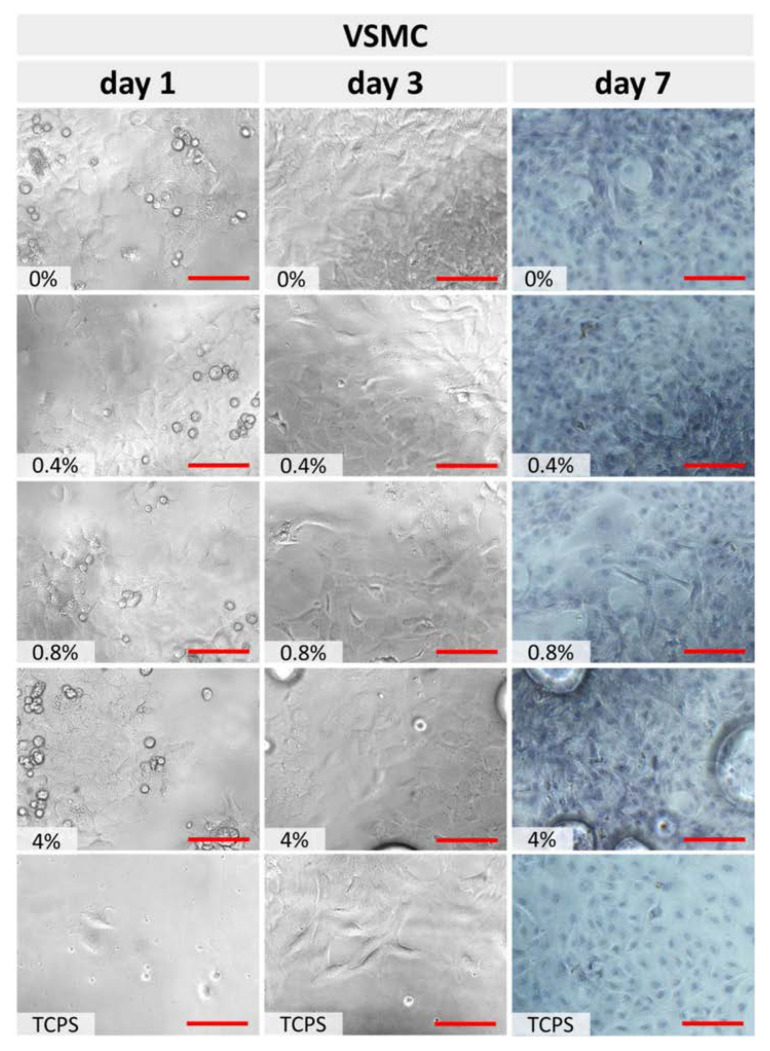
Optical microscopy of VSMCs after 1, 3 and 7 days of culture on an unmodified cPOC surface (0 wt.% of GSH) and on cPOC modified with different concentrations of GSH (0.4, 0.8 and 4 wt.%). Cells cultured on TCPS served as a control. After day 7, the cells were stained with hematoxylin and eosin. Scale = 200 μm (red line).

In turn, Figure 6 represents VSMCs cultured on cPOC samples. The cells grew evenly over the surface, forming a typical “hills and valleys” pattern, in which they overlapped each other, and individual cells were poorly distinguishable, especially in later culture intervals. VSMCs densely covered the entire cPOC materials, leaving practically no free space.

**Figure 7:**
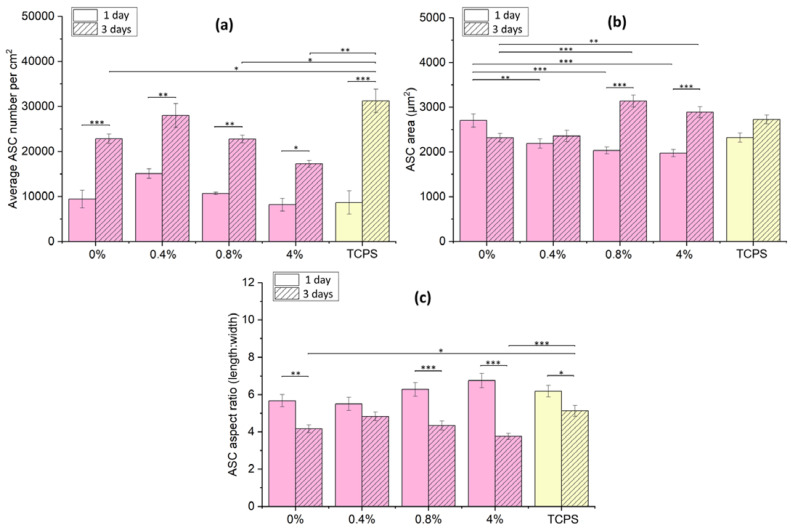
ASCs cultured for 1 and 3 days on unmodified cPOC (0 wt.%), on cPOC modified with different concentrations of GSH (0.4, 0.8 and 4 wt.%) and on TCPS: average cell number per cm^2^ (**a**), average cell spreading area (**b**) and cell aspect ratio (**c**). Mean ± SE, statistically significant differences at * *p* < 0.05, ** *p* < 0.01, and *** *p* < 0.001.

A comparison of the cell numbers on the tested materials (Figure 7) showed that the ASCs rapidly proliferated, as indicated by significant differences on each sample between days 1 and 3 of culture. The cell number achieved on day 3 was usually lower in comparison with TCPS, with the exception of the cPOC_GSH_0.4 sample, where the cell number was similar as on TCPS. The cell spreading area of ASCs cultured on cPOC was also similar to that on TCPS. However, in comparison with cPOC, the cell area decreased in accordance with the increasing amount of GSH on day 1. In turn, the cell area increased greatly on day 3 on the cPOC_GSH_0.8 and cPOC_GSH_4 samples. The cell aspect ratio (length to width) of cells cultured on all materials was within a similar, rather wide range between 4:1 and 6:1, which means that the cells were rather elongated (this is equivalent to the standard morphology for this cell phenotype). On day 3, in general, the cell aspect ratio decreased, but the biggest change could be observed for cPOC and cPOC_GSH_4 in comparison with TCPS. Apart from the cPOC_GSH_0.4 sample, where the ASCs developed most comparably with TCPS, and on which a higher cell number was observed, the remaining amounts of GSH significantly affected the cell morphology (changing it from spindle-like to a more polygonal shape) and had a rather negative effect on the cell numbers.

## 4. Discussion

In this study, an attempt was made to modify the POC polymer by adding GSH. It was found that as the addition of GSH increased, the value of the water contact angle also increased. The surface character of the prepared materials changed from slightly hydrophilic to hydrophobic (as evidenced by an increase in the water contact angle from about 80° for the control cPOC samples to almost 100° in the presence of 8 wt.% of GSH). In turn, the addition of GSH resulted in a decrease in the surface free energy values.

GSH, which is naturally present in the human body, was chosen as a POC modifier for its antioxidant properties. A DPPH• free radical scavenging assay was carried out in order to check whether the inclusion of this substance into cPOC would provide it with antioxidant properties. Free radicals are present in a number of reactions that take place in cells. Sometimes, the level of reactive oxygen species increases, but the cell mechanisms are capable of fighting them. However, when the concentration level of free radicals is too high, cells are subjected to stress, which often causes damage to DNA or to proteins, and leads to the cell apoptosis. On the basis of the obtained results, it can be concluded that the addition of GSH to cPOC provided it with good antioxidant properties, which were improved by an increasing concentration of GSH inside the material. Therefore, GSH-modified cPOC can be a potential solution for reducing the oxidative stress in blood vessels.

In the next step, unmodified and modified cPOC were evaluated in direct contact with ASCs and VSMCs, which are suitable candidates for the reconstruction of the blood vessel *tunica media*, in order to select the optimal concentration of GSH for providing the material with antioxidant properties. These properties would support regeneration of the blood vessel tissue, would counteract the formation of free radicals and would protect the vessel against lumen blockage caused by the adhesion and activation of immune cells and thrombocytes, followed by inflammation and thrombus formation.

Most of the studies reported in the literature on the medical applications of cPOC materials focus on using a 1:1 molar ratio of the substrates, which has in the past been assessed as the most favorable [13]. However, the cell culture studies described in our previous work showed that a 1:1 ratio of the substrates caused significant acidification of the culture medium and, consequently, resulted in a cytotoxic effect on the cells [30]. However, cPOC with a molar ratio of 2:3 did not exhibit such behavior. In the work presented here, there was also no observed decrease in pH (based on visual observation of the color of the cell culture medium). Our results indicated that the studied cPOC materials with a molar ratio of 2:3 supported the adhesion and considerable subsequent proliferation of ASCs and VSMCs. A higher concentration of GSH resulted in dark brown coloration of the cPOC, which made optical microscopy observation of the surface difficult (4 wt.%) or impossible (8 wt.%, therefore not presented here).

Both studied cell types (ASCs and VSMCs) cultured on cPOC with a low content of GSH, particularly 0.4 and 0.8 wt.%, confirmed the lack of cytotoxicity of both the polymer and the GSH modifier. However, the cytotoxicity studies of the materials were burdened by limitations of the viability tests—live–dead (calcein and propidium iodide) fluorescence staining and the resazurin reduction-based Alamar Blue test—which had been performed in our previous studies [30]. Preliminary studies showed that, after the cells on cPOC had been stained, whether or not GSH modification had been performed and irrespective of the concentration of the GHS, the surface of the material exhibited strong autofluorescence (stronger than the fluorescence of the stained cells). This made it impossible to observe the cells under a fluorescence microscope. The same effect was obtained after attempts to stain the cells with other methods (fluorescence staining for DAPI/phalloidin staining, Hoechst staining). However, the presence of GSH in cPOC affected the results of the Alamar Blue test, as it generated a spontaneous reduction of resazurin, even if the cells had earlier been washed off from the surface. Thus, the resazurin reduction values could incorrectly suggest the presence of a richly developed and metabolically active cell culture on the surface of cPOC. Other tests, e.g., MTT and XTT, based on a similar principle, also failed.

Having limited room to maneuver, we decided to observe the cells (in their native state or after staining with hematoxylin and eosin) directly, using optical microscopy, and to perform a thorough analysis of their morphology. When the cell culture medium for the ASCs was replaced on day 3 after seeding, TGFβ1 and BMP4 were added to the medium. It is known that these two members of the transforming growth factor-beta superfamily are able to cause the differentiation of stem cells towards smooth-muscle cells [34]. The short duration of the experiment did not allow for full ASC differentiation, and it was also not possible to verify changes in cell morphology using fluorescence staining (due to problems with the autofluorescence of cPOC). However, the increased cell spreading area, i.e., the cell–material contact area (apparent on materials with 0.8% and 4% of GSH), and the enhanced intercellular contact observed by optical microscopy in the present study, are important factors known to stimulate the cell differentiation and phenotypic maturation of cells (for a review, see [37]). These results suggest that the addition of GSH into cPOC is not only able to support cell viability and growth, but also provides an environment suitable for the initiation of the differentiation process.

VSMCs formed a wide uniform layer on the cPOC surface after 1, 3 and 7 days of culture. The fact that we successfully cultured two different cell types on the same material, including VSMCs, which play a key role in the regeneration of blood vessels, as they are located in the *tunica media* of the vessel, means that cPOC shows great potential for application in vascular substitute development and in vascular tissue engineering. It is important that performing cell culture on these materials did not require pre-coating with adhesive proteins, such as fibronectin or laminin, for ASCs and VSMCs. These pre-treatment steps are often considered necessary to achieve adhesion of the cells to the biomaterial. It is therefore a significant advantage that even unmodified cPOCs are able to support cell adhesion without additional treatment. Nevertheless, the adhesion of the cells could be further improved by physical treatment of the cPOC-based material, e.g., by plasma treatment, in order to increase its hydrophilicity and to activate its surface for potential grafting of various bioactive molecules [34].

## 5. Conclusions

It can be concluded that the modification of cross-linked poly(1,8-octamethylene citrate) (cPOC) biomaterials with glutathione (GSH) has been demonstrated to be effective. The presence of GSH in the material was confirmed by the FTIR-ATR technique. The addition of GSH increased the water contact angle of the material surface (cPOC with 0.4 and 0.8 wt.% of GSH) and slightly lowered the values of its surface free energy. This indicates that the material with a small addition of GSH, in general, maintained its good physicochemical properties after modification. The DPPH• free radical scavenging assay results demonstrated that increasing the concentration of GSH provides cPOC with better antioxidant properties, making it a potential solution for reducing the oxidative stress in blood vessels.

Cell studies with adipose tissue-derived stem cells and vascular smooth muscle cells showed an advantage of adding 0.4 and 0.8 wt.% of GSH to the cPOC, as these additions not only did not impede cell colonization, but even had a beneficial effect on it. Higher concentrations of GSH (i.e., 4 and 8 wt.%) triggered an unfavorable reaction to the materials, which lost their transparency, and their surface took on a hydrophobic character. Studies on adipose tissue-derived stem cells in a differentiation medium have demonstrated that the addition of GSH into cPOC supported cell viability and growth, and that this also provided an environment suitable for the initiation of cell differentiation. Our studies have confirmed that GSH-modified cPOC has suitable physicochemical properties and is cytocompatible with moderate growth of the two cell types. Thus, our results indicate the potential of GSH-modified cPOC materials for producing small-diameter blood vessel grafts.

## Figures and Tables

**Table 1:** Identifiers of the cross-linked polymers unmodified or modified with GSH.

Sample Identifier	Amount of GSH Water Solution (50 mg/mL) per 100 mL of 30% *w/v* Prepolymer Solution	Amount of GSH Water Solution (100 mg/mL) per 100 mL of 30% *w/v* Prepolymer Solution	Percentage of Modifier (wt.%)
cPOC	-	-	0
cPOC_GSH_0.4	2.5 mL	-	0.4
cPOC_GSH_0.8	-	2.5 mL	0.8
cPOC_GSH_4	25 mL	-	4
cPOC_GSH_8	-	25 mL	8

## Data Availability

Data presented in this study are available upon request from the corresponding author.
